# Dexmethylphenidate-associated hemorrhagic bullous IgA vasculitis

**DOI:** 10.1016/j.jdcr.2026.03.011

**Published:** 2026-03-13

**Authors:** Milan Mahesh, Manju Thomas, Mikael Horissian

**Affiliations:** a Drexel University College of Medicine, Philadelphia, Pennsylvania; b Department of Pediatrics, The Wright Center for Graduate Medical Education, Scranton, Pennsylvania; c Department of Dermatology, Geisinger College of Health Sciences, Danville, Pennsylvania

**Keywords:** bullous dermatosis, dexmethylphenidate, drug reaction, IgA vasculitis, pediatric vasculitis

## Introduction

IgA vasculitis (IgAV), formerly Henoch–Schönlein purpura, is the most frequent systemic vasculitis of childhood, with an annual incidence of 3 to 27 per 100,000 children.^1^ Although it can occur at any age, the incidence in adults is very low with over 90% of cases occurring in children. The peak incidence is between the ages of 4 and 6 years.^1^^,^^2^ It has a slight male preponderance. Classic features include palpable purpura, arthralgia, abdominal pain, and renal involvement.^1^^,^^2^ While most cases are self-limited, bullous and necrotic variants are rare and can lead to scarring.^3^ We present a case of hemorrhagic bullous IgAV temporally associated with dexmethylphenidate use in a pediatric patient with attention deficit hyperactivity disorder (ADHD).

## Case report

**Fig 1:**
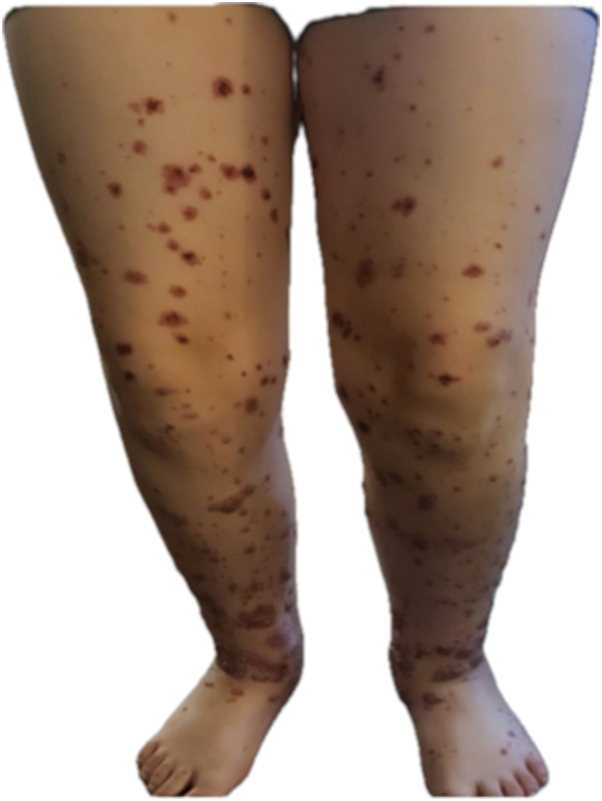
Violaceous papules and plaques in bilateral legs.

**Fig 2:**
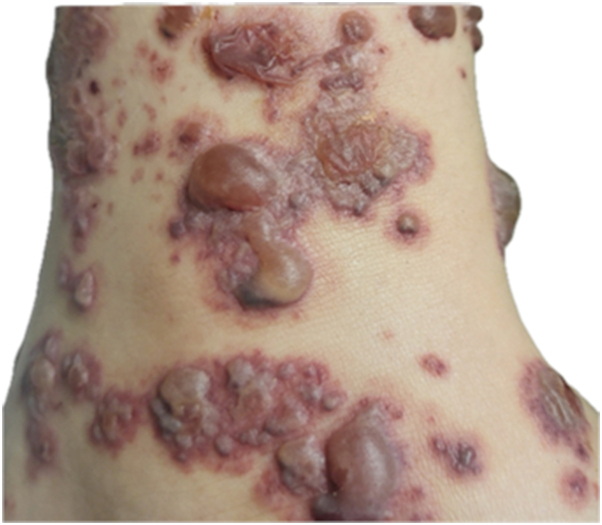
Hemorrhagic tense bullae in lower leg.

A 9-year-old boy with ADHD developed painful violaceous papules and plaques on both legs (Fig 1) 2 weeks after initiating dexmethylphenidate. These progressed to become tense hemorrhagic bullae within 3 days (Fig 2). Lesions extended to the buttocks and upper limbs, impairing ambulation.

Initial outpatient treatment with antihistamines and topical corticosteroids was ineffective. On hospital admission, vital signs were stable. Laboratory studies revealed normal complete blood count and inflammatory markers, with mild proteinuria. Serologic testing for infectious (streptococcal, viral, mycoplasma) and autoimmune (Antinuclear Antibody, Antineutrophil Cytoplasmic Antibodies, complement) etiologies was negative.

A punch biopsy of a thigh lesion demonstrated leukocytoclastic vasculitis with IgA deposition on direct immunofluorescence, confirming the diagnosis of IgA vasculitis. High-dose intravenous methylprednisolone followed by weekly methotrexate led to complete resolution; however, the lower extremity lesions healed with severe scarring and hyperpigmentation.

Dexmethylphenidate was initially discontinued but later reintroduced following multidisciplinary discussion. The eruption recurred within 1 week of rechallenge, prompting the clinical team to recommend permanent discontinuation. The patient improved with a short course of oral corticosteroids.

## Discussion

IgAV diagnosis requires palpable purpura plus at least one of the following: abdominal pain, arthritis/arthralgia, renal involvement, or histopathologic evidence of IgA-predominant leukocytoclastic vasculitis.^4^^,^^5^

Bullous lesions occur in <2% of IgAV cases and are associated with prolonged healing and residual scarring.^6^ Differential diagnoses include linear IgA bullous dermatosis, bullous pemphigoid, and erythema multiforme. Skin biopsy with direct immunofluorescence is essential for diagnosis.^6^

Drug-induced IgAV is rare but increasingly recognized. Pharmacovigilance analyses have identified vaccines, antibiotics, and TNF-α inhibitors as common culprits.^7^^,^^8^ The pathogenesis likely involves immune complex deposition in small vessels.^8^ Psychostimulants, including methylphenidate and dextroamphetamine, have been linked to vasculopathic syndromes such as Raynaud phenomenon and necrotizing vasculitis.^9^ One prior case from Turkey reported methylphenidate-induced IgAV.^10^

Our patient’s temporal relationship between dexmethylphenidate initiation, rash onset, improvement after drug withdrawal, and recurrence upon rechallenge strongly supports dexmethylphenidate-induced hemorrhagic bullous IgAV.

## Conclusion

It is important to maintain a broad differential diagnosis in pediatric bullous eruptions. Skin biopsy and direct immunofluorescence are essential for diagnostic confirmation of IgA vasculitis. When diagnosis is uncertain, drug-induced etiologies should be considered even for medications not previously associated with IgAV. Bullous IgAV may require systemic corticosteroids and immunosuppressants for control. In some cases, it may be difficult to definitively diagnose a drug as the culprit; and in such cases rechallenge can be considered cautiously on a case-by-case basis, with close monitoring and discontinuation promptly upon any sign of recurrence.

Our case report describes a rare bullous form of IgA vasculitis temporally related to dexmethylphenidate therapy in a child. Awareness of potential psychostimulant-associated vasculitis is critical as ADHD medication use continues to rise.

## Conflicts of interest

None disclosed.

## References

[1] Oni L., Sampath S. (2019). Childhood IgA vasculitis (Henoch-Schönlein purpura)—advances and knowledge gaps. Front Pediatr.

[2] Piram M., Maldini C., Biscardi S. (2017). Incidence of IgA vasculitis in children: a population-based study. Rheumatology.

[3] Castañeda S., Quiroga-Colina P., Floranes P. (2024). IgA vasculitis: an update on treatment. J Clin Med.

[4] Ruperto N., Ozen S., Pistorio A. (2010). EULAR/PRINTO/PRES criteria for Henoch–Schönlein purpura: part I. Ann Rheum Dis.

[5] Ozen S., Pistorio A., Iusan S.M. (2010). EULAR/PRINTO/PRES classification criteria: part II. Ann Rheum Dis.

[6] Alonso de la Hoz J., Martínez Antequera C.E., Fernández Manso B. (2021). Hemorrhagic bullous IgA vasculitis: prognosis. Reumatol Clin (Engl Ed).

[7] Piram M., Mahr A. (2013). Epidemiology of IgA vasculitis. Curr Opin Rheumatol.

[8] Rasmussen C., Tisseyre M., Garon-Czmil J. (2021). Drug-induced IgA vasculitis: a pharmacovigilance-based approach. Autoimmun Rev.

[9] Syed R.H., Moore T.L. (2008). Methylphenidate and dextroamphetamine-induced peripheral vasculopathy. J Clin Rheumatol.

[10] Kucukdag M., Unal N. (2023). Methylphenidate-induced Henoch-Schönlein purpura: a case report. Mathews J Case Rep.

